# Autosomal Recessive Retinitis Pigmentosa Associated with Three Novel *REEP6* Variants in Chinese Population

**DOI:** 10.3390/genes12040537

**Published:** 2021-04-07

**Authors:** Lujia Zhang, Ya Li, Litao Qin, Yu Wu, Bo Lei

**Affiliations:** 1 Graduate School, Xinxiang Medical University, Xinxiang 453003, China; lujiazhang61@126.com; 2 Henan Clinical Research Center for Ophthalmic Diseases, Henan Branch of National Clinical Research Center for Ocular Diseases, Henan Eye Institute, Henan Eye Hospital, Zhengzhou University People’s Hospital, Henan Provincial People’s Hospital, Zhengzhou 450003, China; liyadyx@163.com; 3 Henan Medical Genetics Institute, Henan Provincial Key Laboratory of Genetic Diseases and Functional Genomics, People’s Hospital of Zhengzhou University, Zhengzhou, Henan 450003, China; litao_qin@163.com; 4 Shanghai Flash Interpretation Biotechnology, Shanghai 201615, China; nana.wu@tgexsanyi.cn

**Keywords:** *REEP6*, autosomal recessive retinitis pigmentosa, variant, next generation sequencing, protein stability

## Abstract

Retinitis pigmentosa 77 is caused by mutations of *REEP6* (MIM: 609346), which encodes a protein for the development of photoreceptors. Our study was to identify disease-causing variants in three Chinese families using targeted next-generation sequencing (NGS). Multiple lines of computational predictions combined with in vitro cellular experiments were applied to evaluate the pathogenicity of the newly found variants. Three novel variants in *REEP6*, including one missense variant, c.268G>C, one frameshift variant, c.468delC, and one splicing variant, c.598+1G>C, were found, while c.268G>C was detected in all probands. The three variants were classified as likely pathogenic by the American College of Medical Genetics and Genomics (ACMG). *REEP6* variant proteins c.268G>C and c.468delC in cultured cells destabilized the REEP6 protein and induced intracellular inclusions. Our data suggested that *REEP6* c.268G>C may be a recurrent causative variant in Chinese autosomal recessive retinitis pigmentosa patients.

## 1. Introduction

Retinitis pigmentosa (RP) is the most common form of inherited retinal degeneration (IRD), typically starting with night blindness [1]. Visual acuity may be normal but constricted visual field occurs in the early stages [2]. RP is characterized by the degeneration of photoreceptors with autosomal-dominant, autosomal-recessive, and X-linked modes of inheritance as well as rare mitochondrial and digenic forms. Eighty-nine RP related genes have been identified (RetNet, https://sph.uth.edu (accessed on 18 February 2021)). Ophthalmic examinations coupled with next-generation sequencing (NGS) is currently the most commonly used technique for the diagnosis of RP.

RP 77 is caused by mutations of receptor expression-enhancing protein 6 (*REEP6* (MIM: 609346)), which encodes a protein for the development of photoreceptors (NCBI, https://www.ncbi.nlm.nih.gov/gene/92840 (accessed on 18 February 2021)). *REEP6* is a member of the *REEP* family, involved in intracellular trafficking by controlling cargo capacity at the endoplasmic reticulum (ER) [3]. Previous studies demonstrated that loss of *REEP6* in mouse and zebrafish (*REEP6^-/-^*) resulted in progressive retinal degeneration [4,5,6,7]. Agrawal et al. found that *REEP6^-/-^* mice presented progressive photoreceptor degeneration from P20. The ER of rod photoreceptors was expansive, the number of mitochondria increased, and the expression of guanylyl cyclases, GC1 and GC2, could hardly be detected in the outer segment of mouse retina. They considered that the absence of the REEP6 protein prevented vesicles carrying GC1 and GC2 from being transported from the ER to the outer segments of the photoreceptors. The absence of GC protein resulted in a reduction in cGMP in the retina, which kept the cGMP gated ion channels closed. Therefore, rod cells remained in a hyperpolarized state leading to ER stress and abnormal increasing of mitochondria, accumulatively contribute to the cell death [4]. Another study indicated that REEP6-marked clathrin-coated vesicles transported specific cargo to the STX3-resident plasma membrane in rod photoreceptors [5].

Ten causative variants in *REEP6* associated with autosomal recessive retinitis pigmentosa have been reported, including five missense variants, two frameshift deletions, one duplication, one splicing variant, and one genomic rearrangement [5,7,8,9,10]. All probands from the ten unrelated families showed characteristic retinitis pigmentosa. Among these cases, only one family was from China. Here we reported three novel variants in *REEP6* in three probands from unrelated Chinese RP families, and evaluated the pathogenicity of the variants.

## 2. Materials and Methods

### 2.1. Subjects and Clinical Examinations

This study adhered to the Declaration of Helsinki and was approved by the Ethics Committee of Henan Eye Hospital (HNEECKY-2019(15)). Informed consents were obtained from all the participants. Three affected individuals from three families (family A, B, C) were enrolled for ophthalmic examinations including visual acuity, slit-lamp, fundus photography, fundus autofluorescence (AF), swept-source optical coherence tomography (SS-OCT, VG200D SVision Imaging, Luoyang, China), visual field, and full-field electroretinography (ff-ERG).

### 2.2. Targeted NGS and Sanger Sequencing

DNA was extracted from venous blood for genotyping. Genomic DNA of the three probands were subjected to NGS based on a custom-designed panel (PS400), containing 376 genes associated with posterior segment eye diseases, including the exons and adjacent intron regions (50 bp) and known intron mutations [11]. Captured DNA was eluted, amplified and purified, and sequenced using an Illumina high-throughput sequencer. The average sequence depth of the whole exome was 300×, and 99% of the target sequence was over 30×. Raw reads were mapped to the human genome reference (UCSC hg19) via XYGeneRanger 2.0 (Xunyin, Shanghai, China), TGex (LifeMap Sciences, Alameda, CA, USA), and Efficient Genosome Intepration System, EGIS (SierraVast Bio-Medical Technology Co., Ltd, Shanghai, China). Sanger sequencing was performed to confirm the variants, and family segregation was performed in family members when available.

### 2.3. In Silico Analysis

Allele frequencies of the variants in different populations were determined using Exome Aggregation Consortium (ExAC, http://exac.broadinstitute.org/ (accessed on 18 February 2021)), 1000 Genomes Project (1000G, http://browser.1000genomes.org (accessed on 18 February 2021)), Genome Aggregation Database (genomeAD, http://gnomad.broadinstitute.org/ (accessed on 18 February 2021)), and dbSNP (http://www.ncbi.nlm.nih.gov/SNP (accessed on 18 February 2021)). Functional consequences through different in silico algorithms were available via VarCards [12] including CADD (model: GRCh38-v1.6), Mutation Taster, SIFT, PolyPhen2, GERP++, phyloP, phastCons, et cetera. Splice site changing was predicted by NetGene2 [13] and NNSplice [14]. Nucleotide conservation was predicted by GERP, phyloP, PhastCons, and SiPhy via VarCards. Amino acid sequences between species were contrasted by Clustal Omega [15]. The protein structure was predicted by I-TASSER online 3D structure prediction server. Domains were predicted by CDD online software [16]. Project HOPE [17] and ProtParam [18] were used to predict the structural changes by the variants. Stability of proteins encoded by the variants was predicted by MUpro [19] and I-Mutant v2.0 [20]. Pathogenicity of the variants was assessed according to the standards and guidelines published in 2015 by the American College of Medical Genetics and Genomics (ACMG) [21].

### 2.4. REEP6 Expression Constructs

Coding sequence of *REEP6* (NM_001329556.3), *REEP6* c.268G>C, and *REEP6* c.468delC combined with HA tag was synthesized and cloned into pcDNA3.1 vector separately by Sangon Biotech (Shanghai, China). The constructed vectors were confirmed by Sanger sequencing (Sangon Biotech).

### 2.5. Cell Culture and Transient Transfection

HEK293T cells were purchased from American Type Culture Collection (ATCC, Manassas, VA, USA). The cells were cultured in high glucose DMEM medium (SH30022.01, HyClone, UT, USA) with 10% fetal bovine serum (35-081-CV, Corning, NY, USA), 100 U/mL penicillin (Solarbio, Beijing, China), and 100 U/mL streptomycin (Solarbio). The cells were incubated in a humidified incubator at 37 °C in 5% CO_2_. Constructed expression plasmids were transfected into HEK293T cells using Lipofectamine 3000 (L3000015, Invitrogen, CA, USA) at the amount of 2.5 μg/well, in 6-well plates plated at 300,000 cells/well.

### 2.6. Real-Time PCR

At 24 h after transfection, total RNA was extracted from the cells using TRIzol reagent (Ambion, CA, USA) according to the manufacturer’s instructions. Complementary DNA (cDNA) was generated using the PrimeScript RT reagent kit (RR037A, Takara, Dalian, China). Real-time PCR was performed by ABI Prism 7500 system (Applied Biosystems, Thermo Fisher Scientific, MA, USA) using the following primers: F: 5’-GTCACTCTGCTAAGCCTGTATC-3’, R: 5’-GCTCTCGATAGCTTTGATTGAG-3’, in a total volume of 20 μL using Power UpTM SYBR Green Master Mix (Thermo Fisher Scientific). Relative quantification was achieved by the comparative 2^−ΔΔCt^ method as described previously [22]. Student’s t-test was applicated for analysis.

### 2.7. Western Blotting

At 20 h after transfection, HEK293T cells transfected with pcDNA3.1, HA-*REEP6*, HA-*REEP6* c.268G>C, and HA-*REEP6* c.468delC were treated with 100 μM cycloheximide (CHX, HY-N0901, MedChemExpress, NJ, USA) dissolved in DMSO. Cells were scraped at 0, 7, and 10 h after CHX exposure using ice-cold PBS. Cells were centrifuged, and lysed by RIPA lysis buffer (PC101, EpiZyme, Shanghai, China) including 1% protease inhibitor cocktail (GRF101, EpiZyme). After brief sonication, cell lysate was collected. The protein concentration was determined with BCA protein assay kit (P0011, Beyotime, Shanghai, China). All samples were boiled for 5 min at 100 °C, diluted by 5X SDS loading buffer (LT101S, EpiZyme). Equal amounts of protein were loaded on a 12.5% polyacrylamide gel for SDS-PAGE electrophoresis and transferred to PVDF membranes (Millipore, Burlington, MA, USA). PVDF membranes were blocked in 5% skim milk prior to incubation with mouse anti-HA (1:500; 2367S, Cell Signaling Technology, Beverly, MA, USA) and rabbit anti-β-actin (1:1000; 4970S, Cell Signaling Technology). After 2 h of incubation with secondary antibodies at room temperature, the membranes were visualized on a Bio-Rad ChemiDoc MP Imaging System. β-Actin was used as loading control.

### 2.8. Immunofluorescence

At 24 h after transfection, cells were treated for 2 h with 50 μM MG-132 (in DMSO). Cell slides were fixed with 4% paraformaldehyde (in PBS) for 30 min at room temperature (RT), and permeabilized with 0.5% Triton X-100 (in PBS) for 20 min after washing with PBS. Slides were incubated with 5% bovine serum albumin at room temperature for 2 h and then with mouse anti-HA (1:100; Cell Signaling Technology) and rabbit anti-calnexin (1:100; Cell Signaling Technology) for 8 h at 4 °C, respectively. After three washes in PBS, donkey anti-mouse IgG-AlexaFlour 488 antibody (1:200; abs20014, Absin, Shanghai, China) and donkey anti-rabbit IgG-AlexaFlour 594 antibody (1:200; abs20021, Absin) was applied for 2 h. Nuclei were labeled with DAPI (in PBS) for 5 min. Cell slides were imaged using two-photon microscopy (Zeiss NLO 780, Carl Zeiss, Oberkochen, Germany).

## 3. Results

### 3.1. Clinical Examinations

**Figure 1:**
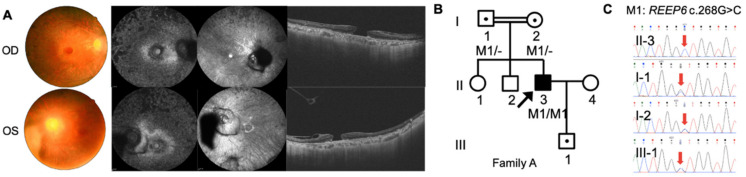
Phenotype and genotype of family A. A homozygous mutation of *REEP6* c.268G>C was found in the consanguineous family. (**A**) Clinical findings of proband A: II-3 including fundus photography, short wavelength AF, IR images and SS-OCT images. (**B**) Pedigree of family A. Black arrows represented the proband. Double lines represented consanguineous. (**C**) Sanger sequencing confirmation of individuals from family A.

**Figure 2:**
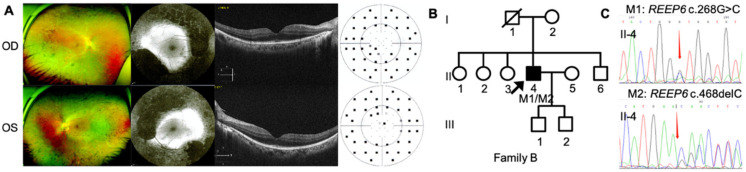
Phenotype and genotype of family B. Heterozygous variants of *REEP6* c.268G>C and c.468delC were found in this family. (**A**) Clinical findings of proband B: II-4 including fundus photography, AF and SS-OCT images. (**B**) Pedigree of family B. Black arrows represented the proband. (**C**) Sanger sequencing confirmation of proband B: II-4.

**Figure 3:**
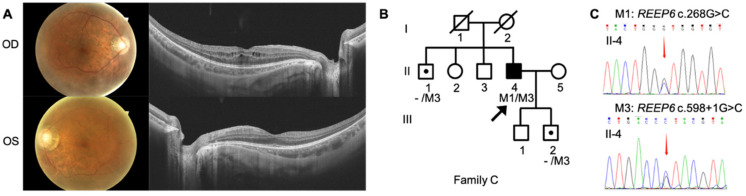
Phenotype and genotype of family C. Heterozygous variants of *REEP6* c.268G>C and c.598+1G>C were found in this family. (**A**) Clinical findings of proband C: II-4 including fundus photography and SS-OCT images. (**B**) Pedigree of family C. Black arrows represented the proband. (**C**) Sanger sequencing confirmation of proband C: II-4. Sanger sequencing confirmation of other individuals from family C was shown in Supplementary Figure S1.

The three Chinese RP pedigrees followed an autosomal recessive inherited trait (Figure 1, Figure 2 and Figure 3). Clinical characteristics were shown in Table 1. Proband A: II-3 from a consanguineous family manifested constrictive visual fields and night blindness in his 30s. Visual acuity reduced to hand movement in his both eyes within 5 years in his 50s. On the latest follow-up, his visual acuity was no light perception in the right eye and light perception in the left eye. Fundus showed bone-spicule deposits. Outer nuclear layer and ellipsoid zone loss outside the fovea was observed, with evident bilateral macular holes and epiretinal membranes in SS-OCT (Figure 1). ff-ERG responses were non-recordable in both eyes. Proband B: II-4 was a 51-year-old man with night blindness and tunnel visual fields for 30 years. Best corrected visual acuity was 0.6 in the right eye and 0.4 in the left eye. Typical bone-spicule deposits in fundus photography and tunnel visual fields in both eyes were observed (Figure 2). ERG responses were severely decreased in both eyes. Proband C: II-4 was a 44-year-old man with night blindness for five years. He came to our hospital because of decreased visual acuity and tunnel visual fields for half a year. Visual acuity was 0.5 in the right eye and 0.4 in the left eye. Fundus photography showed narrowed retinal vessels. SS-OCT showed cystoid macular edema in his right eye (Figure 3).

### 3.2. Mutation Analysis

A total of sixty-nine variants in the three probands were detected. Most of the detected variants were ruled out based on the characteristics of the pedigree and phenotype. Benign or likely benign variants predicted to be neutral or tolerated by multiple lines of computational evidence were excluded.

For proband A: II-3, homozygous variants *REEP6* c.268G>C, p.Val90Leu, and *NPHP4* c.3160C>T, p.R1054C (NM_015102.3) remained after filtering. *NPHP4* is a candidate gene of nephronophthisis (NPH) and Senior–Løken Syndrome (SLS). Since the proband presented only RP without kidney disorders, the biallelic variants in *REEP6* was the only candidate RP-associated variants. The parents and the son of the proband, who did not present any signs, are heterozygous carriers of this variant, while his wife did not have such a variant (Figure 1).

Proband B: II-4 is heterozygous for two variants of *REEP6* c.268G>C, p.Val90Leu and c.468delC, p.Asn156Lysfs*14 remained as the only candidate causative variants after filtering, confirmed by Sanger sequencing (Figure 2).

Proband C: II-4 is heterozygous for two variants of *REEP6* c.268G>C, p.Val90Leu and c.598+1G>C were selected as the causative variants after filtering. Individuals C: II-1 and C: III-2 are heterozygous carriers of c.598+1G>C, confirmed by Sanger sequencing (Figure 3 and Supplementary Figure S1).

**Figure 4:**
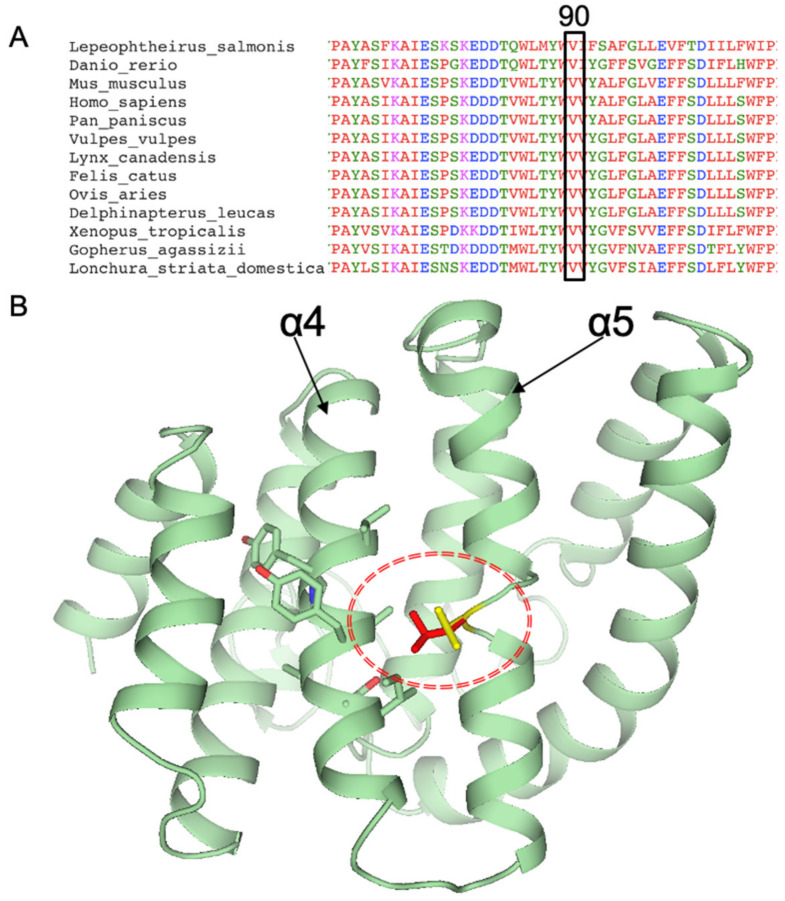
(**A**) Amino acid sequences of REEP6 in 90 loci between 13 species. (**B**) Protein structures encoded by *REEP6* wild type and c.268G>C, p.Val90Leu (Val in yellow, Leu90 in red).

*REEP6* c.268G>C was found in all three probands. Allele frequency in gnomAD was 0.0012 in all populations and 0.0111 in the East Asian population. Allele frequencies in other databases and different populations were shown in Supplementary Table S1. Most of the bioinformatics analysis including CADD, Mutation Taster, SIFT, PolyPhen2, et cetera, indicated that the missense variant was damaging or probably damaging (Supplementary Table S2). Nucleotide conservation predictions by GERP, phyloP, PhastCons, and SiPhy were conservative. Amino acid sequences of REEP6 in 90 loci between species were highly conservative (Figure 4A). Protein structure of REEP6 p.Val90Leu was predicted to be changed (Figure 4B). Since Val and Leu are both hydrophobic residues and the side chain of Leu is bigger than Val, mutation from Leu to Val most likely forms steric hindrances between α4 and α5 helices of REEP (shown by a dashed oval). The mutant residue was bigger than the wild type, which was located in a transmembrane domain. The changed size may affect the contacts with the lipid-membrane (Supplementary Table S3). ProtParam, MUpro, and I-Mutant all indicated that REEP6 p.Val90Leu was instable compared to the wild type (Supplementary Table S3).

*REEP6* c.468delC was not found in ExAC, dbSNP, and 1000 Genomes databases. It was predicted to be disease causing by Mutation Taster (score: 1) and damaging by CADD (score: 22.3). The one-nucleotide deletion in exon 4 led to a frameshift, which results in a premature stop codon. Exons 5 and 6 were lost. ProtParam showed a decreased molecular weight and an increased instability index (Supplementary Table S3), suggesting an incomplete protein structure.

*REEP6* c.598+1G>C was not found in ExAC, dbSNP, and 1000 Genomes databases. Splice donor site changed was predicted by NetGene2 and NNSplice.

### 3.3. Variants in REEP6 Affected the Stability of the Encoded Proteins

**Figure 5:**
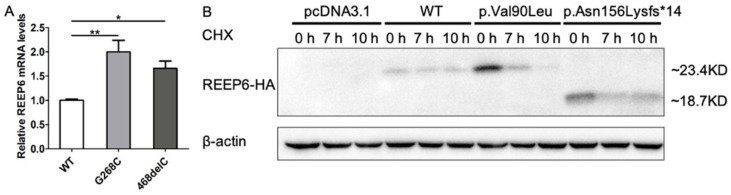
(**A**) The mRNA expression of *REEP6* detected by real-time PCR. Relative expression was normalized to β-actin (* *p* < 0.05, ** *p* < 0.01, *n* = 3). WT = wild type. (**B**) Protein stability evaluated by Western blot. β-actin was used as loading control.

**Figure 6:**
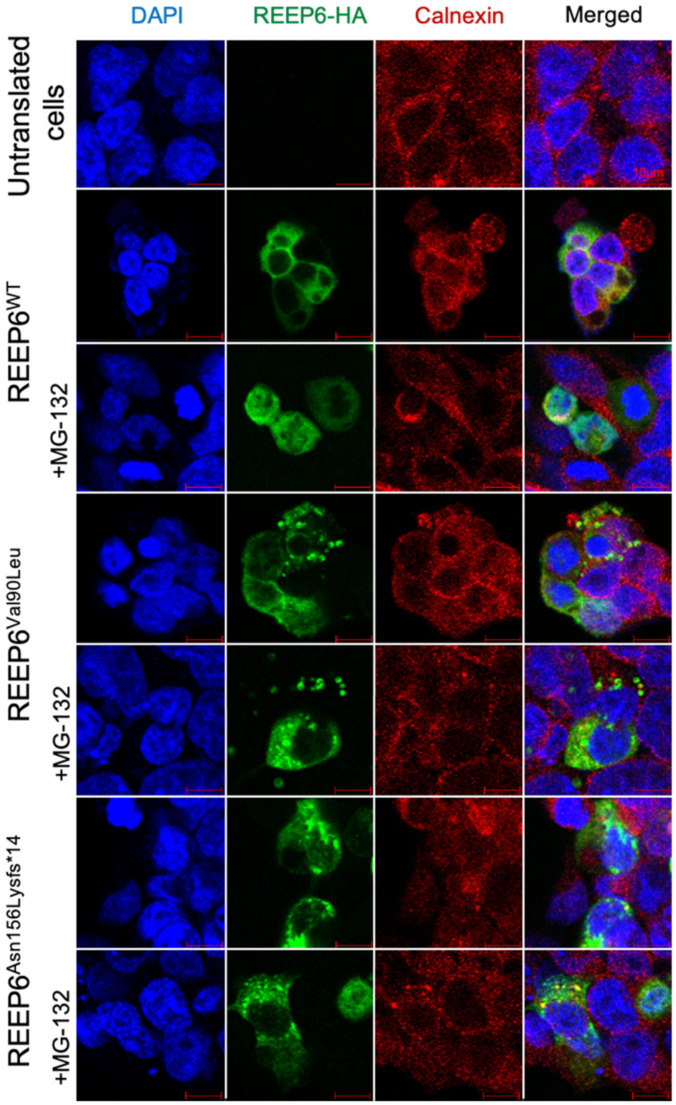
The expression of wild type, p.Val90Leu and p.Asn156Lysfs*14 REEP6 proteins in HEK293T cells. Cells were treated with MG132 for 2 hours before fixation. REEP6 were labeled with anti-HA (green). ER was labeled with anti-calnexin (red). DAPI (blue) was used to stain nuclei.

**Figure 7:**
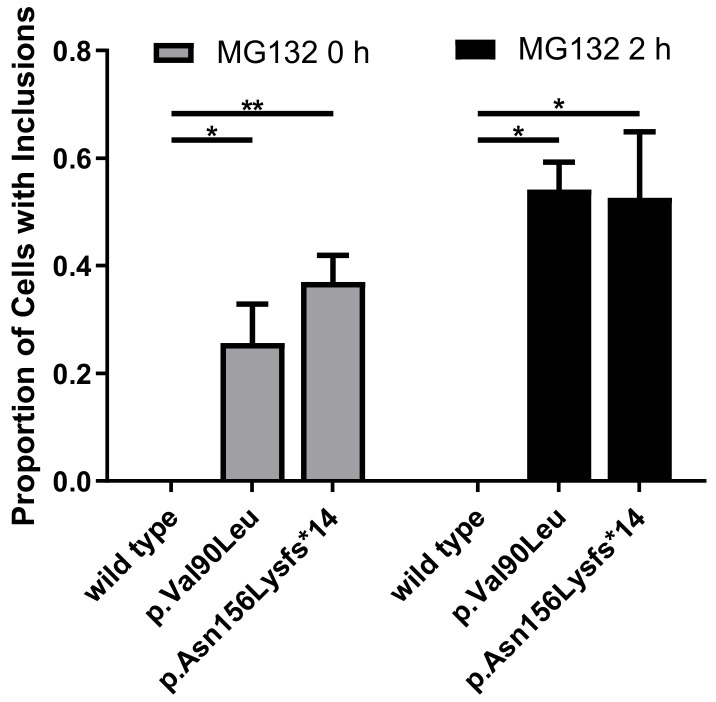
Translated cells with inclusions compared to those without (* *p* < 0.05, ** *p* < 0.01, *n* = 3).

To examine whether the variants c.268G>C and c.468delC affected the protein expression, wild type and mutated human *REEP6* with HA tag were constructed and transfected into HEK293T cells. Relative mRNA levels of the two variants, detected by real-time PCR, were significantly increased compared to the wild type (*p* < 0.05, *p* < 0.01) (Figure 5A). Moreover, CHX was used to inhibit protein synthesis at 24 h after transfection. Our data indicated that p.Val90Leu and p.Asn156Lysfs*14 degraded faster than the wild type, suggesting a significantly shortened protein half-life compared to that of the wild type (Figure 5B). Localization of the two mutated proteins were predominantly colocalized with ER as the wild type. However, intracellular inclusions, a sign of dysfunctional protein aggregates, were seen (Figure 6). Differences of translated cells with inclusions compared to those without in wild type and mutant groups had statistical significance (Figure 7).

## 4. Discussion

We identified three novel variants in *REEP6* including one missense, one frameshift, and one splicing. According to ACMG standards and guidelines, the three variants were classified as likely pathogenic. Supported evidences are listed in Supplementary Table S4.

Notably, proband A: II-3 also has a homozygous variant in *NPHP4*. Mutations in *NPHP4* result in NPH and SLS. NPH is an autosomal recessive cystic kidney disease leading to end-stage renal disease at a median age of 13 years [23]. A small percentage of NPH patients have extrarenal manifestations that constitute a recognizable syndrome such as SLS. SLS is characterized by NPH and RP [24], which is already symptomatic during childhood. We also noticed that a mutation in *NPHP4* was considered to be the causative for RP in Wang’s study [25]. However, the age and diagnosis of the patients were not given. It is hard to know whether the mutation in that research caused non-syndromic RP or syndromic RP. As for proband A: II-3 in our study, RP onset was in his 30s and visual acuity decreased quickly in his 50s. Furthermore, physical examination showed no renal disease. Therefore, we suggest although the variant in *NPHP4* was not the main cause of RP in proband A, there was still a possibility that the mutation in *NPHP4* might contribute to RP.

**Figure 8:**
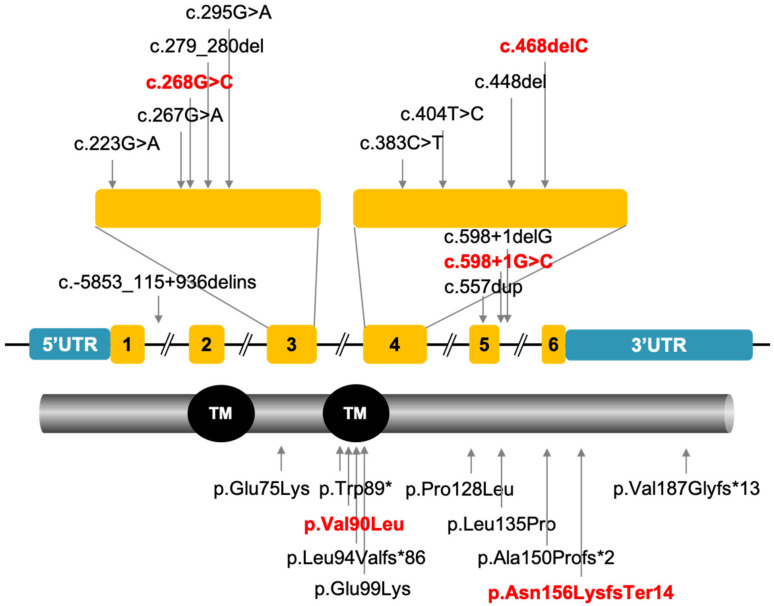
Ten variants in *REEP6* leading to RP have been reported. The novel variants found in this study are shown in red.

To date, only ten variants in *REEP6* have been reported in RP patients (Figure 8) [5,7,8,9,10]. Nyctalopia and gradually constricted visual field were initial symptoms, followed by reduced visual acuity, in all affected individuals in the ten families. Fundus photography showed narrowed retinal vessels and bone-spicule deposits. OCT images showed thin outer nuclear layers in most individuals. In our study, typical manifestations of RP were observed in probands A: II-3 and B: II-4. In comparison, the symptom of proband C: II-4 was moderate, as only tunnel vision and cystic macular edema were observed. He was diagnosed as retinitis pigmentosa sine pigment.

Among the ten variants reported previously, only *REEP6* c.404T>C, p.Leu135Pro knock-in mice have been produced. Homozygous knock-in mice were viable and fertile. Although *REEP6* ^c.404T>C^ mice presented less severe ocular symptoms than *REEP6^-/-^* mice, the mutation in *REEP6* caused photoreceptor degeneration and dysfunction. Significant thinning of the outer nuclear layer and reduced rows of nuclei were observed in mutant mice at 6 months. Full-field scotopic ERGs showed reduction of both a- and b-waves. Phenotypes of the homozygous knock-in mice were similar to the clinical phenotypes of RP patients caused by this mutation [7].

*REEP6* c.268G>C, p.Val90Leu was identified in one homozygous variant and two heterozygous variants in this study. We also noticed that this variant was retrieved as benign in the Netherland population in the Leiden Open Variation Database [26]. Nevertheless, evidence we have collected supports c.268G>C likely being pathogenic in this cohort of patients.

Firstly, in silico analysis and the functional experiments indicated the variant is harmful. The phenotypes and genotypes of the probands are consistent. Multiple lines of computational predictions support that *REEP6* c.268G>C was damaging. Nucleotide and amino acid conservation were predicted to be conserved. REEP6 p.Val90Leu was indicated to be instable, which was also demonstrated by our laboratory experimental data. This variant was in the putative transmembrane domain in REEP6. The mutant residue was bigger than the wild type, which may affect the protein contacts with the lipid-membrane. In addition, we noticed that it was also located in the TB2_DP1_HVA22 domain (66–143 amino acid) which was important in modulating specific G protein-coupled receptor (GPCR) trafficking by affecting ER cargo capacity (https://www.ncbi.nlm.nih.gov/Structure/cdd/wrpsb.cgi (accessed on 18 February 2021)). In fact, six of the ten causative variants are located in this domain, suggesting the significance of this domain in maintaining the function of REEP6 protein. The altered amino acid was predicted to affect the contacts with the lipid-membrane, which may disrupt interactions between REEP6 and the ER membrane. Thus, targeted transport of cargo in transduction of visual signals in photoreceptors might be disrupted [5]. It was defined as likely pathogenic by the ACMG. Furthermore, we observed intracellular inclusions in transfected HEK293T cells. This was consistent with Chen’s research which found the same phenomenon in SK-N-SH cells transfected with *REEP6* c.404T>C and c.383C>T, respectively [7]. The formation of intracellular inclusions in mammalian cells was often as a result of active, retrograde transport of misfolded proteins [27,28]. Dysfunctional REEP6 protein might eventually lead to the degeneration of photoreceptors.

Secondly, whole exome sequencing was also performed on proband A: II-3. We did not find other causative variants in all known genes associated with retinal diseases. Additionally, in another RP 77 family from Peking Union Medical College Hospital, *REEP6* c.268G>C was also defined as one of the causative compound heterozygous variants (Ruifang Sui, personal communication).

However, we cannot explain the individuals with homozygous *REEP6* c.268G>C variant in gnomAD. There is a possibility that he might be in the early stage of RP, or his macular was not affected yet. His central vision might still be sufficient for his normal life, and he was not aware of this. Indeed, the visual acuities of the two probands in the advanced stage of RP were 0.4–0.6 in this cohort of patients.

We also noticed that allele frequencies of this variant were 0.0111 in East Asians and <0.0012 in other populations in gnomAD (Supplementary Table S1). In our IRD database, the frequency of this variant is 0.015 in 165 samples including a homozygous *REEP6* in an Usher syndrome patient. He was a 11-year-old boy suffered hearing loss in addition to RP. Best corrected visual acuity was 0.3 in the right eye and 0.2 in the left eye. He was compound heterozygous for two causative variants of *USH2A*. The pathogenicity of *REEP6* c.268G>C in this patient is still under investigation. It is possible that *REEP6* c.268G>C contribute more to the vison loss when the patient gets older. In our non-ocular diseases database, the frequency is 0.009 in 380 samples with no homozygous. The high frequency might be due to the fact that RP caused by this variant is nonfatal. Patients experiencing minor visual symptoms usually do not see a doctor until their vision is significantly affected after their forties. Thus, this variant is passed on to the next generation and leads to a high frequency in the population. A high frequency does not support this variant to be pathogenic. There is still a possibility that it may be a single nucleotide polymorphism. However, based on the above pathogenic evidences, we predict that such a chance is limited. It is true that in-depth studies in animal models are required to further evaluate the impact of this variant.

*REEP6* c.468delC, p.Asn156Lysfs*14 in proband B: II-4 leads to a frameshift variant and a premature termination codon (PTC) encoding a truncated protein. This variant might cause nonsense-mediated decay (NMD) as predicted by Mutation Taster which means no protein would be produced. However, we detected the truncated protein in HEK293T cells indicating this PTC variant might evade NMD in patients. However, because our expression construct only contained the cDNA of *REEP6* and NMD depended on the presence of both exons and introns, whether this PTC variant evaded NMD in vivo could not be determined. In fact, many disease-causing PTC variants were confirmed to evade PTC detection and mRNA degradation to some extent [29,30,31]. NMD-escaping PTC variants might retain partial function or result in more severe phenotypes than those loss-of-function variants [32].

The transcript of *REEP6* expressed in photoreceptors had an additional C-terminal helix, potentially binding specific interacting proteins or mediating interactions between the ER and mitochondria [3,7]. The PTC was in exon 4 of *REEP6*, resulting in the absence of exon 5, exon 6, and the C-terminal helix. We also noticed that another two frameshift variants nearby leading to the absence of exon 5, exon 6, and the C-terminal helix, were reported to be causative [7]. Moreover, intracellular inclusions were also found in cells transfected with *REEP6* c.468delC. Based on these results, we predicted that REEP6 c.468delC caused photoreceptor degeneration.

*REEP6* c.598+1G>C alters the canonical splice donor site. Intron 5 retention or exon 6 skipping might be observed in the splicing product, resulting in a change in the structure of exon 5. Exon 5 is predicted to encode a short coil and a long loop which might provide an interaction surface for rod photoreceptor-specific modulation of REEP6 [6]. This change might not only disrupt targeted transport of cargo from ER to cell surface but also affect the interactions between the ER and mitochondria, and cause photoreceptor degeneration. At the same position, another splicing variant *REEP6* c.598+1delG was reported to be pathogenic [10].

## 5. Conclusions

While only ten causative *REEP6* variants leading to RP have been reported, we identified three novel variants including one missense variant, c.268G>C, one frameshift variant, c.468delC, and one splicing variant, c.598+1G>C. The missense c.268G>C variant and the frameshift variant in HEK293T cells destabilized the REEP6 protein. Our data suggested that c.268G>C may be a recurrent causative variant for RP in the Chinese population.

## Figures and Tables

**Table 1:** Clinical characteristics of the probands.

Proband	Diagnosis	Onset Age (y)	Age at Diagnosis (y)	BCVA (OD/OS)	ff-ERG	Funds	Visual Field
A: II-3	RP	35	42	NLP/LP	non-recordable	bone-spicule deposits bilateral macular holes	NA
B: II-4	RP	21	51	0.6/0.4	severely decreased	bone-spicule deposits thin interdigitation zone	tunnel
C: II-4	RP	39	44	0.5/0.4	NA	cystoid macular edema (OD) narrowed retinal vessels	tunnel

Abbreviation: RP = retinitis pigmentosa; y = years old; BCVA = best corrected visual acuity; OD = oculus dextrus; OS = oculus sinister; NLP = no light perception; LP = light perception; ff-ERG = full-field electroretinography; NA = not available.

## Data Availability

Data presented in this study is contained within the article and Supplementary Material.

## Supplementary Materials

The following are available online at https://www.mdpi.com/article/10.3390/genes12040537/s1, Figure S1. Sanger sequencing confirmation of individuals from family C, Table S1: Allele frequency in population of *REEP6* c.268G>C, Table S2: In silico prediction of REEP6 c.268G>C, Table S3: Predicted protein physico-chemical parameters and structural modifications are as shown, Table S4. Supported evidence of three novel variants.
