# Supplementary material for: Autosomal Recessive Retinitis Pigmentosa Associated with Three Novel REEP6 Variants in Chinese Population
Source: Genes (Basel). 2021 Apr 7;12(4):537. doi: 10.3390/genes12040537 (PMC8068040; doi:10.3390/genes12040537)
Supplement: Supplementary file 1 [file genes-12-00537-s001.pdf]

Supplementary

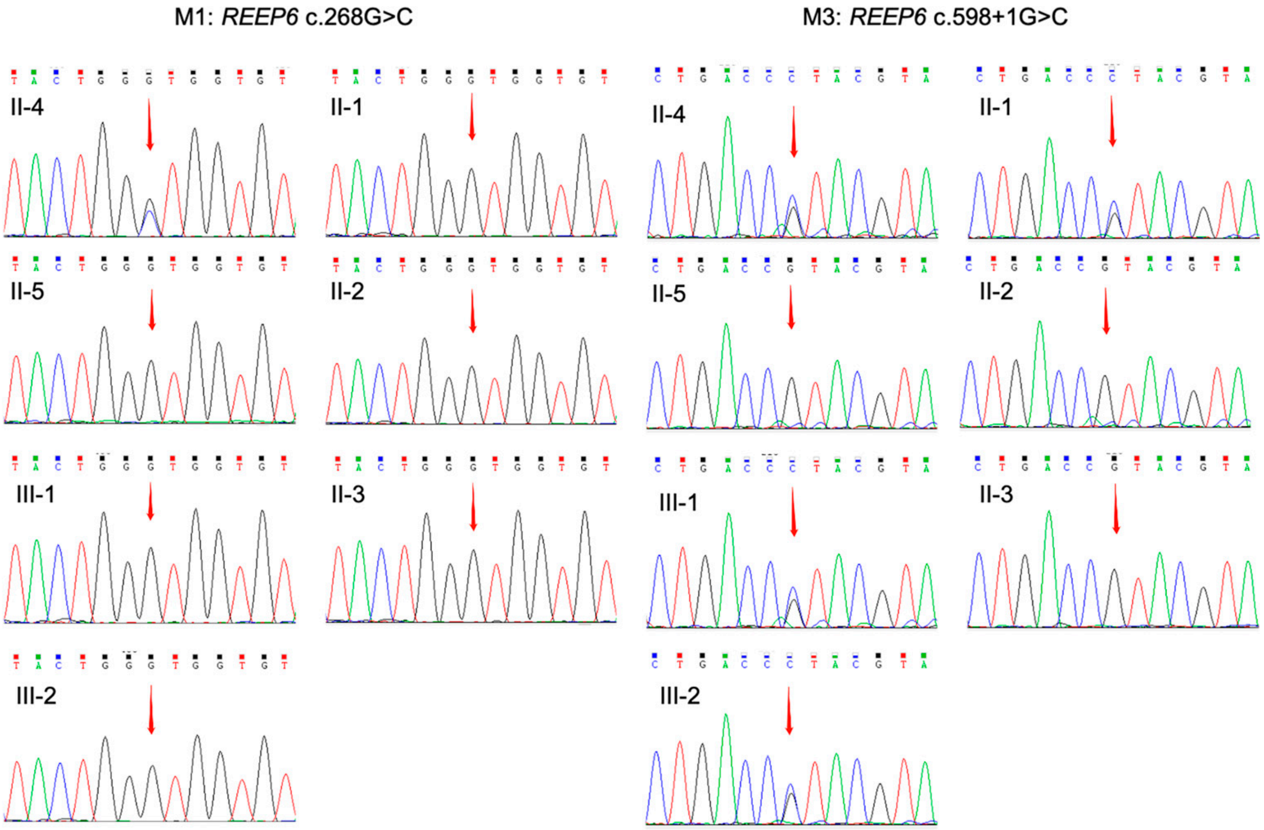

Figure S1. Sanger sequencing confirmation of individuals from family C.

Table S1. Allele frequency in population of *REEP6* c.268G>C.

Database	Homozygous	Allele frequency in population						
		ALL	East Asian	South Asian	African American	Latino	Finnish	Non-Finnish European
1000G	0	0.0046	0.0208	0.001	0	-	-	-
ExAC	0	0.0013	0.0116	0.0010	0	0	0.0014	0.0003
gnomAD exome	1	0.0012	0.0111	0.0012	0	0	0.0011	0.0002
gnomAD genome	0	0.0005	0.0068	-	0	0	0.0009	0.000067
dbSNP	-	0.00022	0.009	0.00	0.0000	0.000	0.00018	

Table S2. *In silico* prediction of REEP6 c.268G>C.

Algorithm	*REEP6* c.268G>C	
	**Score**	**Prediction**
SIFT	0.002	Damaging
Polyphen-2 HDIV	1.0	Probably damaging
Polyphen-2 HVAR	0.999	Probably damaging
Mutation Taster	1	Disease causing
FATHMM	–3.47	Damaging
PROVEAN	–2.92	Damaging
VEST3	0.778	Damaging
MetaSVM	0.744	Damaging
MetaLR	0.867	Damaging
CADD	32	Damaging
DANN	0.998	Damaging
FATHMM MKL	0.971	Damaging
Eigen	0.666	Damaging
GenoCanyon	1.000	Damaging
MutationAssessor	3.33	Medium
fitCons	0.696	Tolerable
REVEL	0.231	Tolerable
ReVe	0.628	Tolerable
ClinPred	0.11271138	Benign
GERP++	4.53	Conserved
phyloP	9.859	Conserved
phastCons	1.000	Conserved
SiPhy	16.28	Conserved

Table S3. Predicted protein physico-chemical parameters and structural modifications are as shown.

Type	Molecular weight	Theoretical pI	Instability index	Structural modification
			**ProtParam/MUpro/ I-Mutant**	
wild type	23418.31	8.74	33.64/–/–	-
p.Val90Leu	23432.34	8.74	34.62/–0.33 (Instable)/ -1.96 (Instable)	The mutant residue is bigger than the wild type, which is located in a transmembrane domain. This changed size may affect the contacts with the lipid-membrane.
p.Asn156fs	19198.46	8.43	35.02/–/–	The mutation leads to a slightly truncated protein, might cause NMD. Exon 5 and exon 6 were deleted in transcript NM_001329556.3.

Table S4. Supported evidence of three novel variants.

Variant	Criterion	Basis	Evidence	Classification
c.268G>C (p.Val90Leu)	PM3	For recessive disorders, detected in trans with a pathogenic variant	To be classified as PM3-Strong based on detection in trans with two pathogenic variants	Likely Pathogenic
	PM1	Located in a mutational hot spot and/or critical and well-established functional domain (e.g., active site of an enzyme) without benign variation	Located in TB2_DP1_HVA22 domain (66–143 amino acid) which is important in modulating specific G protein-coupled receptor trafficking by affecting ER cargo capacity	
	PP3	Multiple lines of computational evidence support a deleterious effect on the gene or gene product (conservation, evolutionary, splicing impact, etc.)	Predicted to be damaging by multiple in silico predictions	
c.468delC (p.Asn156fs)	PVS1	Null variant (e.g., nonsense, frameshift, canonical ± 1 or 2 splice sites, initiation codon, single exon or multiexon deletion) in a gene where LOF (loss of function) is a known mechanism of disease	Frameshift variant and multiexon deletion.	Likely Pathogenic
	PM2	Absent from controls (or at extremely low frequency if recessive) in Exome Sequencing Project, 1000 Genomes Project, or Exome Aggregation Consortium	Absent from controls in 1000G, ExAC and gnomAD	
c.598+1G>C	PVS1	Null variant (nonsense, frameshift, canonical ± 1 or 2 splice sites, initiation codon, single exon or multiexon deletion) in a gene where LOF is a known mechanism of disease	To be classified as PVS1-Strong based on splice doner site changing	Likely Pathogenic
	PM2	Absent from controls (or at extremely low frequency if recessive) in Exome Sequencing Project, 1000 Genomes Project, or Exome Aggregation Consortium	Absent from controls in 1000G, ExAC and gnomAD	
